# Prevalence of interpersonal violence in sports clubs in Germany

**DOI:** 10.3389/fspor.2026.1701609

**Published:** 2026-02-26

**Authors:** Teresa Greither, Sophia Mayer, Thea Rau, Bettina Rulofs, Marc Allroggen

**Affiliations:** 1 Department of Child and Adolescent Psychiatry/Psychotherapy, University Hospital Ulm, Ulm, Germany; 2 German Center for Mental Health (DZPG), Partner Site Ulm, Ulm, Germany; 3 Institute of Sociology and Gender Studies, German Sports University Cologne, Cologne, Germany

**Keywords:** athletes, prevalence, prevention, sports, violence

## Abstract

**Introduction:**

Distressing reports from research and practice illustrate the vital need for protection against interpersonal violence (IV) in sports. IV is defined as the intentional use of force or power between individuals and includes psychological, physical and sexual violence, and neglect. This study aims to illuminate the scope of this issue within German sports clubs by determining the prevalence of IV, both within and outside the sporting context, and to identify risk factors.

**Methods:**

The cross-sectional online survey included questions about personal and sports-related demographics as well as experiences of IV within and outside the sports context. The questionnaire was developed based on existing measures of IV (including IVACS-Q and IVIS). The convenience sample consisted of 4,367 current or former members of German sports clubs (response rate 16.1%). Mean age was 41.4 years (*SD* = 17.6), and athletic level ranged from recreational to international, thus representing the full range of sport club members. Descriptive statistics were used to assess the prevalence of IV, and chi-squared tests, phi correlations, and logistic regression were used to determine associations between IV inside sports and gender, age, athletic level, early specialization, and experiences outside of sports.

**Results:**

A majority (70.0%) of participants reported at least one experience of IV within a sports club. A large overlap with experiences outside of sports (59.0%) was found. Psychological violence was the most prevalent form (63.0%). All forms of IV were closely intertwined, especially psychological violence, which often co-occurred with other forms of IV. IV experiences outside of sports were strongly related to IV inside sports. Individual and sports-specific characteristics such as, gender or athletic level, partially proved to be risk factors for some forms of IV.

**Discussion:**

Our findings suggest that sports club members are affected by IV, even at lower levels of performance and across all age groups. Results indicate that experiences of IV inside and outside sports are related, implying an increased risk of revictimization in either context. Individual and sports-specific characteristics need to be discussed as differentiated risk factors to improve prevention.

## 1 Introduction

High-profile cases of interpersonal violence (IV) in international competitive sports – such as the sexual abuse scandals in gymnastics in the US (1) or the Rubiales case in Spanish soccer (2) - have significantly heightened public and academic attention to the issue. These incidents have triggered debates about the extent of IV and the failure of current preventive measures, highlighting structural deficiencies in organized sports. In Germany, previous studies have similarly identified deficits in safeguarding practices within sports organizations. These studies emphasize the central role of community-based sports clubs, which integrate athletes of all ages and competitive levels into the same organizational structure, and in which safeguarding measures must address diverse needs and risk profiles (3). Despite growing awareness, most existing research on IV in sports has focused on youth and elite-level athletes, often overlooking experiences that occur in adulthood (4). This has left the experiences of the broader club sports population largely unexamined. However, IV is not confined to specific performance levels or age groups. It occurs across all sporting contexts and affects athletes in various disciplines and life stages (5–7).

### 1.1 Defining and categorizing IV in sports

IV encompasses a broad spectrum of harmful behaviours. In the literature, terms such as abuse, maltreatment, harassment, and bullying are often used interchangeably, despite reflecting distinct disciplinary perspectives, theoretical frameworks and relational dynamics (5–8). This lack of conceptual clarity obscures the scope of the issue and complicates efforts in both research and prevention (5). To address this, the study uses a clear and inclusive definition of IV, drawing on the World Health Organization (WHO) framework, which also underpins the 2024 International Olympic Committee (IOC) consensus statement on IV and safeguarding in sport (5, 9): Violence is defined as “the intentional use of (physical) force or power, threatened or actual, against oneself, another person, or against a group or community, that either results in or has a high likelihood of resulting in injury, death, psychological harm, maldevelopment or deprivation” (9), thereby including a wide range of actions which may lead to adverse physical or psychological outcomes. IV encompasses the sub forms of psychological, physical, sexual violence, and neglect. The term “interpersonal” refers to the relational nature of the violence, indicating that it occurs between two or more individuals (as opposed to e.g., self-directed violence), and in the context of sports manifests itself between athletes, coaches, or other stakeholders such as administrators or chaperones (5, 9).

Despite some variability in scholarly perspectives, several conceptualizations agree that certain behaviors should be considered harmful, specifically within the context of sports (5, 6, 8, 10). Based on previous studies (6, 10–16), the following interpretation of IV was adopted and guided initial questionnaire development:

(1) Psychological violence: Acts that threaten or have the potential to threaten an athlete's mental health, safety, or development. Such acts include threats, verbal abuse, isolation, insufficient support, or promotion of unhealthy, destructive, or antisocial behaviors.
(2) Physical violence: Actions that lead to actual or potential physical harm to the athlete, including physically aggressive behaviors (e.g., hitting) and acts that may harm the athlete physically (e.g., forced training through injuries). Not included in this definition are all acts part of or carried out within the rules of a sport.
(3) Sexual violence: Acts of a sexual nature, carried out without consent, or despite incapacity to consent Sexual violence is subdivided into two categories:
  (a) Non-contact sexual violence (NCSV) includes behaviors that resemble sexual harassment (e.g., sexualized comments) and acts such as voyeurism, which occur without physical contact between victim and perpetrator. This specifically includes online harassment (phone/internet, videos etc.).
  (b) Contact sexual violence (CSV) includes sexual behaviors involving physical touch or any other form of body contact.
(4) Neglect is defined as the omission of basic care regarding health, education, and safety, especially for children or dependents.

To ensure conceptual clarity and international comparability, this study also addresses recent positions and definitions from the 2024 IOC consensus statement (5). Regarding the distinction of psychological and physical violence, a discrepancy in definitions was detected: Acts that may harm an athlete physically (to force an athlete to train with injuries, engage in unhealthy eating behavior, consume doping products, or punishment with excessive exercise) are defined as psychological violence in the IOC consensus statement, but were categorized as physical violence in this study – following one of the largest studies in the field by Hartill et al. (16).

### 1.2 Prevalence and patterns of IV

A growing body of prevalence studies, which were synthesized in the recent IOC consensus statement, underscores the widespread nature of IV in sport (5, 11–14, 16–25): reported rates range from 44% (11) to 86% (22) for any form of IV, thereby reflecting the high burden of IV across diverse sporting contexts and countries. However, variation in definitions, sampling strategies, and target populations severely limits the comparability of results between studies. Providing context specific to the German population, for instance, a study by Ohlert et al. (12) in 2015 examined lifetime IV experiences among elite athletes in Germany, Belgium, and the Netherlands. This study included both childhood and adult experiences within the German subsample but excluded non-elite participants. In contrast, Hartill et al. (16), in a 2020 retrospective survey conducted across six European countries (including Germany), focused exclusively on experiences before the age of 18 in organized sport. Despite these methodological differences—particularly regarding the inclusion of non-elite and adult participants—both studies reported similarly high prevalence rates: 75.8% in Ohlert et al.'s (12) elite athlete sample (with 88.5% reported in the German subsample) and 75.0% in Hartill et al.'s (16) European sample (with 77.9% in the German subsample). Comparable high findings specifically for elite and youth sports populations have also been reported in Canada (13, 24).

The IOC consensus statement identifies psychological violence as the most prevalent form of IV in sport, with estimates ranging from 21% to 79% (5, 11–13, 16, 24). It is often normalized or justified as part of high-performance culture (7, 26, 27). Other forms of IV are also highly prevalent in sport and often co-occur with psychological violence (12, 16): lifetime exposure estimates for physical violence range from 4% to 66%, sexual violence estimates range from 0.5% to 78% and neglect prevalence estimates range from 27% to 69% (5). These findings underline the importance of addressing the full spectrum of IV, as the various forms are closely intertwined, share root causes, and have comparable impact (5, 11, 12, 16, 17, 24, 28).

### 1.3 Socioecological determinants and consequences

Several individual and contextual determinants (or risk factors) are associated with IV in sport. On the individual level, athletes identifying as LGBTQ+ (lesbian, gay, bisexual, transgender, and queer+) experience higher levels of IV (psychological, physical and sexual violence) across multiple studies (5, 11). Disabled athletes are shown to have elevated exposure to psychological violence (5, 11). The findings regarding gender and exposure to IV are inconsistent in the reviewed literature, with no clear evidence that one group is uniformly more at risk than another across all forms of IV (5, 12, 16, 21). Elevated exposure to sexual violence in girls/women, and elevated exposure to physical violence for males was corroborated in several publications (5, 22, 24). Regarding contextual factors, several variables linked to high-performance environments are associated with increased risk of IV. Regarding the athletic level, elite athletes, or those competing at the national/international level, show elevated exposure to physical violence and psychological violence (5, 11, 14). Athletes training 16 or more hours weekly also report more psychological violence and physical violence (5, 24). Early sport specialization is linked to increased psychological violence and neglect (24).

Age is less frequently included as an analytical variable in studies on IV in sport, resulting in limited knowledge about its influence. One Canadian study involving participants aged 14–17 found that older adolescents reported more IV experiences (24). Overall, research in this field tends to focus implicitly or explicitly on youth populations, based on the well-founded assumption that they are particularly vulnerable to IV due to developmental stage, social positioning, and structural dependencies within sports settings (7).

While this study focuses on IV in sports, it is important to note that experiences of IV often extend beyond the sporting context. Research indicates that individuals exposed to maltreatment during childhood are at increased risk of re-victimization later in life, reflecting a broader pattern of cumulative adversity (29–31). Similarly, IV experienced in sports has been associated with increased vulnerability to IV in other settings, such as school or family environments (16, 24, 32). These overlaps point to shared structural factors—such as asymmetrical power relations and the normalization of transgressive behaviour—that facilitate IV across different social domains (33, 34). Acknowledging this broader context guides a more comprehensive understanding of IV (35). Thus, to fully grasp the pervasive and complex nature of IV in sport, it is necessary to move beyond examining individual incidents and adopt a comprehensive theoretical lens. Drawing from Bronfenbrenner's ecological systems theory (36) the current IOC consensus statement on IV and safeguarding in sport (5) posits that IV is not merely a result of individual acts but arises from the complex interaction of factors across multiple nested levels, which include the individual/interpersonal, organizational, sectoral, societal and temporal level. Acknowledging this socioecological complexity helps explain why IV manifests across all sporting contexts, ages, and competitive levels and is critical for designing safeguarding strategies. The consequences of IV in sports are far-reaching and can be long-lasting. IV is associated with negative psychological, behavioural, physical, and material/performance outcomes (5). These are resembling the outcomes of child maltreatment in other contexts (28, 37–41). Vertommen et al. (28, 40) reported that moderate and severe IV in sports during childhood is associated with lower quality of life and higher psychological distress in adults. Psychological violence—such as belittling, intimidation, or shouting—has also been shown to negatively affect athletes' mental well-being, performance and motivation (37, 39, 42–44). Such forms of IV are often embedded in routine practices, such as training through pain or exhaustion, and are framed as “necessary for success” (7, 26, 45), which may contribute to long-term injuries (19, 46). These patterns align with findings from outside of sport, where violence and abuse are linked to lasting developmental and psychological harm (47–49). While this study does not assess health consequences directly, the established evidence base highlights the importance of recognizing IV as a serious and persistent issue across the lifespan.

### 1.4 Research objective

In the German context, understanding IV in sports requires attention to the specific features of the national sports system. Sports participation is largely organized through a decentralized, community-based club system that integrates athletes of all ages and competitive levels—from recreational to elite—within shared organizational settings. Adults make up the majority of club members and actively participate in training and competition (50) and over 20% of adults are part of such a club. Most IV cases occur in these clubs—during routine activities and within everyday environments such as gyms, locker rooms, and playing fields—rather than in specialized or isolated settings like sports boarding schools (51). Despite this, most IV research has focused on youth or elite athletes, neglecting the broader population of adult club members. This is a critical gap, as the structural features of club sports may expose athletes of all ages to similar risks. Given the central role of sports clubs in promoting health, physical activity, and social engagement, it is essential to develop context-specific and evidence-based prevention strategies. These efforts must be informed by comprehensive empirical data that reflect the full scope of IV within sports club settings (5, 52).

Thus, the aim of this study is to systematically examine the prevalence, risk factors, and co-occurrence patterns of IV in a cross-sectional sample within the context of German club sports. By focusing on athletes across all competitive levels (from recreational to elite) and a broad age range, as well as by including retrospective lifetime reports of IV experiences, this study examines a gap in the existing literature by providing a more comprehensive understanding of IV in this specific sports setting. In addition, the study considers IV experiences outside of sport, allowing for a more contextualized interpretation of the conditions under which IV in sports occurs. The following research questions are proposed:

- Q1: What is the estimated lifetime prevalence and co-occurrence pattern of psychological, physical, and sexual violence, and neglect among athletes across all competitive levels (recreational to elite) and ages in German club sports?
- Q2: What individual and structural factors (e.g., age, athletic level, weekly training time, early sport specialisation, IV outside of sports) are associated with reporting lifetime experiences of different forms of IV in this German club sport context?

## 2 Methods

### 2.1 Interpersonal violence questionnaire development

The IV questionnaire was developed to assess retrospective lifetime experiences of IV within the German sports club context in a cross-sectional sample. To ensure conceptual clarity and international comparability, its structure and content drew from several sources, including the German version of the *Interpersonal Violence In Sports* (IVIS) questionnaire (11, 53), the German version of the *Interpersonal Violence Against Children in Sports* (IVACS-Q) questionnaire (15, 16) and a Canadian elite sports report (54).

The questionnaire assessed five forms of IV: psychological violence (13 items), physical violence (13), non-contact sexual violence (NCSV, 14), contact sexual violence (CSV, 11), and neglect (7). The psychological violence section retained core behaviors from IVACS-Q and IVIS – such as being humiliated, criticized for appearance or performance, threatened for not participating, or deliberately being ignored/excluded – with only minor rewording. This category also introduced an element on perceived favoritism (“feeling that some athletes were preferred over others”), to capture a nuance not explicitly covered in the original scales (54). The physical violence items closely align with those in the German “SafeSport” study (using the IVIS questionnaire) (12, 53), encompassing a range from shaking, hitting with hand or object, and being knocked down, to more coercive practices. Notably, the new questionnaire incorporated IVACS-Q's coverage of coerced physically harmful training practices: respondents were asked about being forced to train while injured or to perform exercise as punishment, and about pressure to take substances for weight control or performance – items that extend or reinforce the IVIS content on bodily harm and doping. The neglect section was largely adopted from IVACS-Q (e.g., lack of basic care, medical neglect, inadequate supervision or equipment, and being pulled from school for sports), and was expanded with one item addressing ignored safety concerns when attempting new skills [adapted from Kerr et al.'s & Willson et al.'s work (13, 54)], to ensure all aspects of welfare neglect in sports were captured. The CSV items were drawn from both IVACS-Q and IVIS and show strong thematic overlap: they range from uncomfortable close physical proximity and unwanted touching or kissing, to attempted or forced sexual acts (oral, vaginal, or anal). These questions were carefully phrased to distinguish levels of severity (e.g., separating attempted assault from completed acts) and include an item on sexual initiation rituals with physical contact, which appears in both IVACS-Q and IVIS as part of hazing-related abuse. Similarly, the NCSV items integrate content from the validated tools: exposing respondents to lewd sexual comments, catcalls, and sexualized remarks about one's body (elements prominent in IVIS, as well as incidents of indecent exposure (“flashing”), unwanted sexual digital communications, and being coerced to view or produce sexual images (detailed in IVACS-Q). An additional item was added to this category to address disability-specific harassment for those with a disability (e.g., someone taking photos emphasizing an athlete's disability in a sexualized way), reflecting an effort to broaden inclusivity beyond what previous instruments covered. Participants were instructed to report experiences they perceived as negative, harmful, or abusive—either at the time they occurred or upon later reflection. Each of the 58 items was behaviorally specific and assessed via a four-point frequency scale: “never”, “once”, “two to four times”, and “five times or more”. For every item, participants were also asked if the behavior had occurred outside of sports (e.g., at school, in the family), using a binary yes/no format. This additional framing enabled comparisons of IV across contexts. For each respondent who reported experiences of IV in sports, a follow-up set of questions collected detailed contextual information about the incidents; these data are not included in the present publication.

The full questionnaire was reviewed and refined linguistically in consultation with all collaborating project partners (e.g., safeguarding experts, regional sports federations and the German sports youth). The final instrument was comprehensive, aligned with validated international measures, and adapted to reflect the realities of club members in Germany's club-based sports system. The 58 IV questions of the German questionnaire are included in the supplementary materials (Supplementary Material 1). The supplement includes an English translation and mentions the original source of each question.

Content validity was established by adherence to construct definitions and by using items that have been validated in prior studies. For reliability analysis, Cronbach's alpha was calculated to assess the internal consistency of the IV scales. The psychological violence scale demonstrated good internal consistency (Cronbach's *α* = .855), indicating a reliable measurement. The physical violence scale showed acceptable internal consistency (Cronbach's *α* = .701). The NCSV scale exhibited excellent internal consistency (Cronbach's *α* = .914). Similarly, the CSV scale demonstrated good internal consistency (Cronbach's *α* = .812). Finally, the neglect scale showed acceptable to good internal consistency (Cronbach's *α* = .770). Overall, all scales demonstrated satisfactory to excellent reliability, indicating that the instruments used in this study are internally consistent and suitable for further statistical analyses.

The present article focuses on a selected subset of survey variables related to the prevalence and correlates of IV. For each sub form of IV, participants were also asked to identify the most severe incident they experienced. For these incidents, detailed information on age, sport-specific context, perpetrator characteristics, and consequences was collected. These data will be examined in future publications. This selective focus allows for an in-depth presentation of core findings, while maintaining conceptual clarity. An overview over participants age at the time of the subjectively most impactful incidents is given.

### 2.2 Measurement of personal and sports-specific characteristics

Age depicts the participants age at the time of the survey (as opposed to the time of the IV incidents) and was analyzed using grouped categories. Age groups were aligned with the age brackets used in the DOSB's annual club membership audit (50) to facilitate comparisons with national sports participation data. Gender was assessed with three categories (male, female, diverse/other).

Participants were asked to indicate their highest level of athletic participation, based on their involvement in competitions or tournaments. Individuals who engaged in sports solely for leisure without participating in competitions were classified as recreational athletes. Those who had represented their club in club or local competitions were categorized as competing at the club or local level. Participants who had represented their club (or region) in district, federal state or region competitions were assigned to the regional level. Those who had competed at national-level events were categorized at the national level, and individuals who had represented their country (e.g., Germany) in international competitions were classified as international-level athletes. This classification system enabled a comprehensive assessment of competitive experience across the spectrum of German club sports.

Weekly training time was grouped into six categories in the questionnaire to capture varying levels of sports participation intensity: up to 2 h, 2.5–6 h, 6.5–10 h, 10.5–14 h, 14.5–20 h, and more than 20 h per week.

Early sports specialization was measured using a three-question instrument adapted from Parent & Vaillancourt-Morel (24), with the items originally synthesized based on research by LaPrade et al. (55). Participants were asked whether they: (1) had chosen a main sport before the age of 12, (2) had trained that sport for more than eight months per year, and (3) had ceased participating in other sports. Those who responded “yes” to all three items were classified as having specialized early in a single sport.

### 2.3 Participants and recruitment

Active and former members of German sports clubs aged 16 and older were eligible for participation. Participant recruitment was supported by 11 of the 16 state sports federations and the DOSB, which represent key organizational pillars of the German club sports system. They facilitated survey dissemination through their communication channels, e.g., via press releases, email lists, websites, social media (e.g., Facebook, Instagram), and newsletters. Federation-affiliated youth organizations, network meetings, and training workshops were also used for dissemination. The German Sports Youth included the study link in its national newsletter. Participants could access the questionnaire via a dedicated website or directly via a QR code. This convenience sampling strategy was adopted because the decentralized, volunteer-based structure of sports clubs in Germany restricts the practicality of probability-based sampling. Access to clubs and participants relied on existing networks and the willingness of individuals to participate, which was consistent with the organizational realities of the field. Given the constraints of time and resources, this approach allowed for efficient recruitment while acknowledging the limitations inherent in non-probability sampling.

### 2.4 Procedure

Data collection was conducted between April and July 2021 using the Unipark EFS Survey platform. Respondents were informed about the study's aims, data protection policies, and their rights to withdraw at any time. Informed consent was required to proceed. Due to the sensitive content, participants were provided with direct links to professional support services throughout the survey.

Eligibility criteria included being at least 16 years old, current or former membership in a German sports club, and submission of plausible responses.

### 2.5 Ethical considerations

This study complied with the principles of the Declaration of Helsinki. Ethical approval was granted by the Ethics Committee of Ulm University on February 10, 2021 (Approval No. 493/20), and data protection clearance was obtained prior to data collection. Participation was anonymous and voluntary, and all procedures related to informed consent, withdrawal, and participant safety were upheld. Information on support services was prominently provided to mitigate potential distress caused by the survey content.

### 2.6 Statistics

The prevalence of IV was measured with a low threshold (i.e., including all IV experiences) and analyzed descriptively. Gender and age group differences, as well as differences between athletic levels and early specialization, were detected using chi-squared tests. Associations between IV experiences inside and outside of sports contexts were evaluated with phi coefficients. Simple and multiple logistic regression models were created to determine associations between gender, age at the time of the survey, athletic level, early specialization, and experiences of IV outside sports with the five forms of IV experiences inside sports as the respective criteria. The remaining four forms of IV inside sports (besides the criterion), were also entered into the simple and multiple regression models as predictors to explore the interwovenness of the forms of IV. Odds ratios (ORs) and 99% confidence intervals (CIs) are reported. The cross-sectional design permits only the identification of associations. Age, athletic level and other predictors were assessed at the time of the survey rather than at the time of the incidents, limiting the temporal interpretation of the findings. The calculations described above were undertaken using SPSS statistics software. The co-occurrence of forms of IV inside sports was visualized using R statistics (56, 57).

## 3 Results

### 3.1 Sample

Of the 27,115 individuals who accessed the online survey, 4,808 completed it. The response rate after accessing the survey was 17.7%. After applying inclusion criteria—i.e., minimum age of 16, current or former sports club membership, informed consent, and plausibility of data—4,367 responses were retained for analysis. This final sample corresponds to approximately 0.024% of the estimated 18.2 million sports club members aged 16 and older in Germany (50) in 2021. On average, participants required 48 min to complete the questionnaire.

Of the 4,367 participants, 46.3% identified as female, 52.6% as male, and 0.5% reported a different gender identity. The mean age was 41.4 years (*SD* = 17.6). Additional sample characteristics, including information on disability, athletic level, training time, early specialization, and type of sports, are presented in Table 1.

**Table 1:** Study sample characteristics.

Variables	*n* = 4367	
	%	*n*
Mean age	41.4 years (*SD* = 17.6 years)	
Age groups		
16–18 years	8.7%	381
19–26 years	20.2%	882
27–40 years	19.7%	859
41–60 years	34.2%	1,492
61 and older	17.2%	753
Gender		
Female	46.3%	2,024
Male	52.6%	2,295
Other	0.2%	22
Disability		
No	96.5%	4,216
Yes	3.5%	151
Sports club membership		
Current member	96.4%	4,208
Former member	3.6%	159
Athletic level		
Recreational	15.8%	688
Club/local level	35.1%	1,531
Regional level	27.7%	1,210
National level	14.1%	615
International level	5.8%	253
Weekly training time		
<2 h	22.6%	987
2.5–6 h	51.6%	2,239
6.5–10 h	16.3%	711
10.5–14 h	4.4%	192
14.5–20 h	1.9%	83
>20 h	1.1%	47
Early specialization		
No	83.8%	3,533
Yes	16.2%	685
Types of sports performed		
Only individual sports	37.3%	1,631
Only team sports	15.2%	664
Both team & individual	42.7%	1,865

SD, standard deviation.

### 3.2 Prevalence of IV

The prevalence of IV was assessed with a low-threshold measure, that is, including all experiences (details in Table 2). Of the participants, 70.0% had experienced IV inside a sports club, and 70.0% reported experiences outside of sports. The most frequent form of violence within a sports club was psychological violence (62.8%). Physical violence was reported by 37.0%, NCSV by 26.4%, and CSV by 19.2% of the participants. The least frequent form of IV was neglect (15.0%). Outside of the sports context, psychological violence was also the most prevalent form (60.8%), followed by physical violence (36.5%), NCVS (32.6%), CSV (29.6%), and neglect (8.3%). Both forms of sexual violence (CSV and NCSV) occurred more frequently outside of sports than inside sports contexts.

**Table 2:** Prevalence of IV outside and inside sports, and their overlap.

Forms of IV	Outside sports	CI (%)	Inside sports	CI (%)	Overlap inside/outside	CI (%)
Any IV	*n* = 4,367		*n* = 4,321		*n* = 4,321	
% (*n*)	70.0 (3,059)	68.7–71.4	70.0 (3,024)	68.6–71.4	59.0 (2,551)	58.6–61.7
Psych. violence	*n* = 4,242		*n* = 4,190		*n* = 4,190	
% (*n*)	60.8 (2,580)	59.4–62.3	62.8 (2,631)	61.3–64.3	50.5 (2,115)	48.9–52.1
Phys. violence	*n* = 4,277		*n* = 4,124		*n* = 4,124	
% (*n*)	36.5 (1,563)	35.1–38.0	37.0 (1,525)	35.5–38.5	22.5 (928)	21.0–23.7
NCSV	*n* = 4,338		*n* = 4,152		*n* = 4,152	
% (*n*)	32.6 (1,415)	31.2–34.0	26.4 (1,095)	25.0–27.7	18.9 (786)	18.0–20.5
CSV	*n* = 4,341		*n* = 4,111		*n* = 4,111	
% (*n*)	29.6 (1,286)	28.3–30.9	19.2 (790)	18.0–20.4	13.1 (537)	12.3–14.5
Neglect	*n* = 4,346		*n* = 4,092		*n* = 4,092	
% (*n*)	8.3 (361)	7.5–9.1	15.0 (615)	13.9–16.1	5.3 (215)	4.7–6.2

IV, interpersonal violence; CI, confidence interval (95%) with lower and upper limit; Psych. Violence, psychological violence; Phys. violence, physical violence; NCSV, non-contact sexual violence; CSV, contact sexual violence.

### 3.3 Inside and outside sports contexts

There was a substantial overlap in experiences of IV in both settings: 59.0% reported having experienced IV inside as well as outside sports settings. Regarding the experience of different forms of IV in both contexts, psychological violence was reported by 50.5% of respondents, physical violence by 22.5%, NCSV by 18.9%, CSV by 13.1%, and neglect by 5.3% of respondents (see Table 2 for details).

The phi coefficients (see Table 3) reflect strong positive associations between experiences of psychological violence and NCSV inside and outside sports. Moderate correlations exist in the prevalence of physical violence, CSV, and neglect inside and outside sports.

**Table 3:** Phi-coefficients between forms of IV inside and outside sports.

Interpersonal violence inside sports	Interpersonal violence outside sports				
	Psychological violence	Physical violence	NCSV	CSV	Neglect
Psychological violence	.504*	.227*	.220*	.173*	.148*
Physical violence	.314*	.365*	.212*	.162*	.184*
NCSV	.307*	.221*	.478*	.287*	.192*
CSV	.234*	.165*	.316*	.389*	.208*
Neglect	.252*	.212*	.259*	.247*	.389*

IV, interpersonal violence; NCSV, non-contact sexual violence; CSV, contact sexual violence. Phi-Coefficients >.400 indicate a strong association, .399–.300 a moderate association and .299–.200 a weak association.

* *p* < .001.

### 3.4 Co-occurrence of forms of IV inside sports

**Figure 1:**
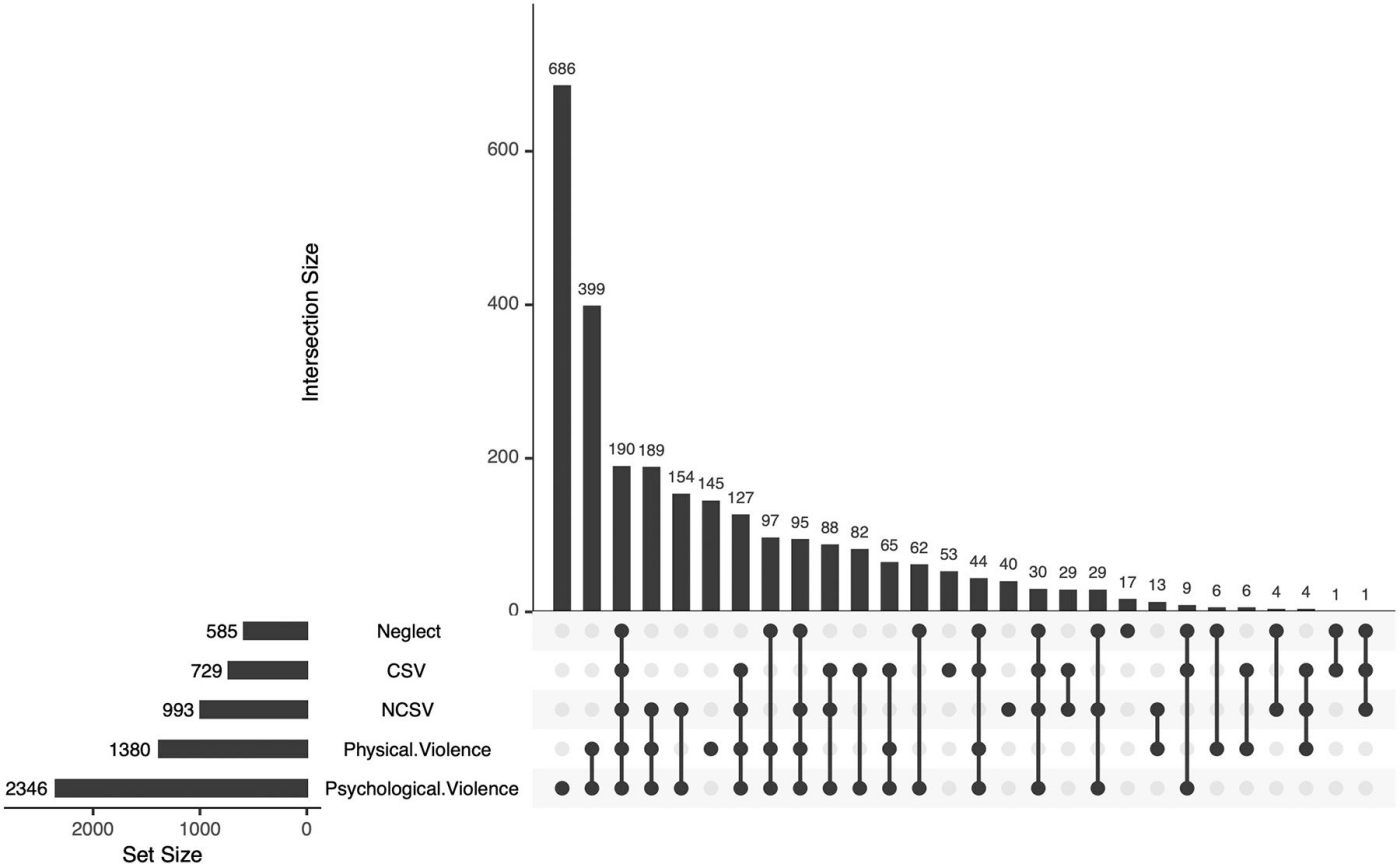
Co-occurrence of all forms of interpersonal violence inside the sports context. NCSV, non-contact sexual violence, CSV, contact sexual violence. This plot displays the total prevalence of each type of IV (set size; left bars) and the size of all observed combinations of IV exposures (intersection size; upper bars). Filled dots in the matrix indicate which forms of IV contribute to each intersection; connected dots represent co-occurring forms of IV. Higher intersection bars reflect more frequent (co-)exposure patterns.

Figure 1 illustrates the co-occurrence of all forms of IV inside sports contexts. The most common was psychological violence only (*n* = 686), followed by the co-occurrence of physical violence with psychological violence (*n* *=* 399), all five forms of violence co-occurring (*n* = 190), and the co-occurrence of NCSV, psychological, and physical violence (*n* = 189). Physical violence, both forms of sexual violence, and neglect were found to co-occur more frequently with psychological violence than to occur as standalone forms. Psychological violence was present in most co-occurring combinations, whereas combinations without psychological violence were comparatively rare.

### 3.5 Gender, age, and sports-specific characteristics

Women (77.1%) experienced significantly more IV inside sports than men (63.6%). A gap in the occurrence of sexual violence (with and without physical contact) of more than 20.0% exists between men and women. Significant differences are also found between the five different age groups: Generally, older age groups (41–60 years, and 60 years or older) reported less IV compared to the youngest age group (16–18 years), across all forms of IV.

Participants could indicate the age at which the subjectively most severe incident occurred for each form of IV: Between 17.7% (physical violence) and 36.2% (sexual violence with body contact) of participants indicated that the incident started after the age of 18. Between 26.5% (physical violence) and 38.7% (NCSV) indicated that the incident ended after the age of 18, and between 7.0% (CSV) and 20.7% (NCSV) indicated that the incident was still ongoing (detailed information on this data will be published separately).

Prevalence also varies significantly between athletic levels, with higher-level athletes reporting more IV. In the same manner, a higher weekly training time was also associated with higher rates of IV. For those participants who had specialized in a single sport before the age of 12 years (early specialization), the prevalence of IV was significantly higher for all five forms of IV, compared to the group of those who had not specialized early. Detailed results are illustrated in Table 4.

**Table 4:** Prevalence of IV inside sports by gender, age, early specialization, and athletic level.

Variables	Forms of interpersonal violence					
	Any IV % (*n*)	Psych. violence % (*n*)	Physical violence % (*n*)	NCSV % (*n*)	CSV % (*n*)	Neglect % (*n*)
Gender						
Women n = 1,907–2,008	77.1 (1,549)	69.2 (1,358)	39.1 (755)	39.1 (759)	31.0 (594)	20.7 (395)
Men n = 2,151–2,273	63.6 (1,446)	57.1 (1,249)	34.9 (755)	14.6 (318)	8.6 (185)	10.0 (215)
χ^2^ (1)	92.800	65.459	7.478	317.641	330.398	90.911
p	<.001	<.001	.006	<.001	<.001	<.001
V	.147	.126	.043	.278	.285	.150
Age						
16–18 years n = 351–373	79.9 (298)	72.5 (261)	50.4 (177)	30.4 (108)	26.4 (92)	25.1 (86)
19–26 years n = 826–873	85.2 (744)	79.5 (667)	54.7 (452)	38.7 (323)	29.3 (245)	23.4 (193)
27–40 years n = 820–857	79.9 (683)	74.1 (617)	45.8 (379)	33.3 (276)	22.1 (181)	17.8 (145)
41–60 years n = 1,407–1,477	63.5 (938)	55.4 (797)	29.5 (419)	22.1 (314)	14.9 (210)	10.7 (151)
61 and older n = 701–741	48.7 (361)	40.1 (289)	14.0 (98)	10.4 (74)	8.8 (62)	5.7 (40)
χ^2^ (4)	341.459	351.662	359.812	194.710	136.404	145.099
p	<.001	<.001	<.001	<.001	<.001	<.001
V	.281	.290	.295	.217	.182	.188
Athletic level						
Recreational n = 652–683	53.4 (365)	44.0 (295)	18.0 (120)	19.1 (126)	13.7 (90)	8.6 (56)
Local n = 1,449–1,521	67.9 (1,033)	60.0 (876)	33.9 (492)	23.8 (350)	18.3 (267)	13.5 (195)
Regional n = 1,128–1,205	77.2 (930)	70.9 (830)	43.9 (502)	29.0 (332)	21.9 (248)	16.0 (181)
National n = 580–613	74.2 (455)	69.7 (415)	45.8 (267)	33.2 (196)	20.7 (120)	20.3 (119)
International n = 235–251	84.1 (211)	75.8 (188)	55.4 (133)	34.4 (83)	24.1 (57)	24.7 (58)
χ^2^ (4)	150.936	168.985	185.791	49.502	23.336	54.793
p	<.001	<.001	<.001	<.001	<.001	<.001
V	.188	.202	.213	.110	.076	.116
Weekly training time						
<2 h n = 936–980	55.7 (451)	47.3 (451)	23.1 (219)	19.2 (181)	13.2 (124)	9.4 (87)
2.5–6 h n = 2,106–2,227	71.0 (1,582)	63.7 (1,367)	37.2 (786)	25.8 (553)	19.1 (405)	13.2 (279)
6.5–10 h n = 670–709	81.1 (575)	74.9 (517)	51.0 (344)	33.3 (225)	24.4 (164)	21.9 (147)
10.5–14 h n = 180–191	84.8 (162)	80.4 (152)	48.9 (88)	40.1 (75)	23.5 (43)	26.8 (49)
14.5–20 h n = 79–83	88.0 (73)	84.1 (69)	57.3 (47)	37.0 (30)	29.6 (24)	35.4 (28)
>20 h n = 44–46	87.0 (40)	78.3 (36)	62.2 (28)	45.5 (20)	38.6 (17)	36.4 (16)
χ^2^ (5)	177.828	188.143	172.267	72.553	51.516	114.484
p	<.001	<.001	<.001	<.001	<.001	<.001
V	.205	.214	.206	.134	.113	.169
Early specialization						
No n = 3,338–3,514	68.0 (2,389)	60.6 (2,059)	34.6 (1,160)	25.6 (864)	18.2 (610)	13.8 (459)
Yes n = 637–682	82.8 (565)	76.3 (516)	53.0 (347)	32.2 (212)	26.0 (168)	23.5 (150)
χ^2^ (1)	60.519	59.656	79.009	12.229	21.034	39.576
p	<.001	<.001	<.001	<.001	<.001	<.001
V	.120	.121	.140	.055	.073	.100

IV, interpersonal violence; Psych. Violence, psychological violence; NCSV, non-contact sexual violence; CSV, contact sexual violence; χ^2^, Pearson's chi-square test, V, Cramer's V (.1 indicates a small, .3 a medium and .5 a large effect). Variation in n-sizes of variables reflects missing data.

### 3.6 Associations between IV experiences, personal characteristics, and sports-specific aspects

Both simple and multiple logistic regressions (see Supplementary Material 2 for detailed results) were used and revealed that gender, age, athletic level, training time per week, early specialization, IV outside of sports, and forms of IV inside sports all contributed to the explanation of between 40.2% and 49.6% of the variance in IV experiences inside sports.

#### 3.6.1 IV outside sports

IV experiences outside sports were found to be strongly associated to IV experiences inside sports for each form of IV. Respondents who reported psychological violence outside of sport had sixfold higher odds of reporting psychological violence within sport compared with those without such experiences outside sport. Similarly, respondents who reported NCSV outside of sport had eightfold higher odds of reporting NCSV within sport. Corresponding associations were observed for CSV (OR = 5), physical violence (OR = 3), and neglect (OR = 6).

#### 3.6.2 IV inside sports

For each of the regression models, one form of IV was used as the criterion, while the remaining four forms of IV inside sports were included as predictors to further explore the interconnectedness of IV inside sports. Overall, all forms of IV inside sports showed significant associations with their respective outcome. Firm relations were found, e.g., when psychological violence was the outcome, as the presence of neglect (OR 4), NCSV (OR 4), physical violence (OR 3) or CSV (OR 2) were all significantly associated with increased odds for psychological violence. For physical violence, NCSV, CSV, and neglect as criteria, a corresponding pattern emerged with psychological violence showing a strong and significant association (ORs ranging from 3 for physical violence to 4 for NCSV, CSV, and neglect). Significant relations between all other forms of IV were detected in each of the multiple logistic regression models.

#### 3.6.3 Personal characteristics

For gender, mixed results emerged for the different forms of IV in the multiple logistic regression models. No significant gender differences could be detected between men and women for psychological violence and neglect. A significant gender difference emerged for physical violence, with men showing higher odds of reporting physical violence (OR=0.7). Higher odds were observed for women for both forms of sexual violence (OR > 2 for NCSV and CSV).

Age group related associations also varied by IV form. No significant age group differences were apparent for psychological violence and CSV. Diverse associations were identified for different age groups regarding the other forms of IV: Older age groups (those above 41 years) showed lower odds of reporting physical violence compared to the youngest age group (16–18 years), whereas the opposite pattern was observed for NCSV, with older age groups showing higher odds of reporting NCSV relative to the youngest group. Another significant association was identified for neglect, with lower odds observed for the age group of 41–60 years.

#### 3.6.4 Sports-specific aspects

The results of the multiple regression models showed heterogenous associations for sports-related aspects. Higher athletic level was associated with significantly higher odds of reporting psychological and physical violence, whereas no relevant associations were observed for NCSV, or neglect. For CSV, a higher OR of 1.5 for the local and regional athletic level was found in comparison to recreational athletes; this association was not observed for the national and international level athletes.

Associations with weekly training time also varied by IV form. Higher odds of psychological violence were observed for those training more than two hours per week; however, those training 20 h or more did not report significantly more psychological violence. For those athletes who were training 14.5 h or more, the odds for neglect were significantly higher in comparison to those training less than two hours per week. For physical violence, those with a training time between 2.5 and 6 h showed higher odds compared to those training less than two hours, while for all other training hours per week, no significant differences were found in the multiple logistic regression. No significant associations were observed between training hours per week and both forms of sexual violence (NCSV and CSV).

Early specialization before the age of 12 in a single sport was included as an additional sport-specific predictor. Early specialization was significantly associated with physical violence. No significant associations were observed for psychological violence, CSV and neglect, while lower odds (OR = 0.7) were detected for NCSV.

## 4 Discussion

This study investigated the prevalence and patterns of interpersonal violence - including psychological, physical, and sexual violence, as well as neglect—among current and former members of German sports clubs, using a cross-sectional self-report survey. Notably, the data span the entire life course, capturing IV experiences that occurred both before and after age 18, and thus provide insights into not only child, but also adult victimization within the sports club context—a population that has been largely overlooked in prior research.

### 4.1 Prevalence and forms of IV

The overall prevalence of IV inside sports was 70%, which aligns with rates reported in other national and international studies (11–13, 16, 24). Although variations in instruments, definitions, and target populations limit direct comparisons, this finding confirms the widespread nature of IV across organized sport. Our study used a comprehensive questionnaire that drew on established tools (11, 12, 16) and incorporated elements from previous surveys (13, 54), enabling comparability as well as the inclusion of newer aspects of IV measurement.

For instance, Hartill et al. (16) found a 75% prevalence rate in a sample from six European countries, based on IV experiences in sports before age 18. In comparison, our slightly lower rate of 70% includes experiences across the lifespan and reflects a broader athletic population. The multinational elite athlete sample studied by Ohlert et al. (12) encompassed experiences that occurred before the age of 18 in the subsample of Dutch/Flemish elite athletes, and all experiences within sports (regardless of age) for the German subsample. Within Ohlert et al.'s report (12), a slightly higher prevalence of 76% for any form of IV than in our study (70%) emerged. Importantly, elite-level athletes are exposed to specific structural risk factors—such as early specialization, longer training hours, and dependencies —which likely contribute to their elevated rates of IV (58). In contrast, our sample included many adult recreational athletes yet still revealed comparably high prevalence. In addition, a substantial proportion of participants reported that their subjectively most severe IV experiences began, ended, or were still ongoing after the age of 18, indicating that IV in sport extends well into adulthood. This highlights that IV is not restricted to elite or youth sports environments.

Our findings on individual forms of IV align with existing research. Psychological violence (63%) was the most common form, consistent with Hartill et al. (16) at 65% and slightly lower than Ohlert et al. (12) at 72%. Psychological violence is often the most common form because it encompasses a broad range of behaviors from overt insults to subtle exclusion, leading to higher reported rates. In addition, it is frequently normalized in sports practice (27, 59). Physical violence was the second most frequently reported form of IV in our sample (37%), which corresponds with Hartill et al. (16) who reported a prevalence of 43.8%. Our prevalence exceeds the elite athlete prevalence from Ohlert et al. (12) who found a lower rate of 24.8%. These discrepancies can be partly attributed to definitional differences, as our questionnaire incorporated items from both studies but applied a broader conceptualization of physical violence. Specifically, certain behaviors that may lead to physical harm—e.g., forced training despite injuries—were classified as physical violence in our study. In contrast, some of these items are categorized as psychological violence or neglect in other instruments, which may account for the observed variation in prevalence. This reflects a broader issue in the field: the lack of consensus on how specific behaviors are classified—as physical, psychological violence, or neglect—poses a challenge for comparability across studies. The IOC consensus statement proposes a clear classification, which could be used in future studies to adapt questionnaires (5).

Comparisons of sexual violence are methodologically challenging, as many studies do not distinguish between contact and non-contact forms of sexual violence, unlike our investigation. This distinction allows for a more differentiated understanding of the nature and extent of sexual boundary violations in sport, compared to studies using aggregated measures. In our sample, 26.4% of respondents reported experiencing NCSV, which is somewhat lower than the 34.6% reported in the European youth athlete sample by Hartill et al. (16) —the only large-scale studies to report separate prevalence rates for CSV and NCSV within organized sport. About one-fifth (19%) of participants reported at least one instance of CSV in our sample, closely aligning with the 20% reported by Hartill et al. In contrast, Ohlert et al. (12) reported a combined prevalence of 30.6% for sexual violence in their elite athlete sample, but their measure did not distinguish between contact and non-contact forms, limiting interpretability.

The comparatively low neglect rate in our study may reflect the adult sample, as neglect is typically more relevant for minors. Differences in how neglect is defined and measured also explain wide variation in previous research [e.g., 37% in Hartill et al. (16); 69% in Willson et al. (13)].

Synthesizing these results, even when IV is assessed across the entire lifespan—as in our study—prevalence rates for most forms of IV remain comparable to those reported in studies surveying youth or elite athletes' experiences, apart from lower rates for neglect. The findings give an indication to broaden both research agendas and safeguarding strategies beyond exclusively youth-focused paradigms (13). While the increased vulnerability of young athletes warrants particular protective measures, the substantial proportion of adult athletes reporting lifetime experiences of IV highlights that many individuals remain affected – either by childhood experiences or by incidents that occurred in adulthood - thus meriting systematic attention in the sports club context. Given that prevention efforts aim to establish safe sports environments through structural and cultural change, it is critical that such initiatives adopt an inclusive approach that encompasses all stakeholders—coaches, administrators, support staff, and athletes across the lifespan. In this context, the inclusion of adult athletes is not only necessary for comprehensive safeguarding but also fundamental to fostering a climate and culture of attentiveness within sports settings.

### 4.2 Comparison of IV inside and outside sports

In our sample, 70% of respondents reported experiencing at least one form of IV, either inside or outside of sport, with a substantial overlap of 59% of participants indicating victimization in both contexts. These findings are comparable to those of Hartill et al. (16), who reported prevalence rates of 75% for IV inside sports and 82% outside sport. In our data, the prevalence of psychological and physical violence did not differ substantially between the sports and non-sports contexts. Consistent with Hartill et al. (16), both forms of sexual violence—NCSV and CSV—were more frequently reported outside of sport. Similar patterns have also emerged in elite athlete samples, where sexual violence was more commonly reported in non-sports contexts than within organized sports environments (32). The prevalence of neglect, however, was higher inside sports than outside. This finding is likely influenced by methodological factors, as the neglect items in our questionnaire were primarily sports specific. Additionally, as discussed above, the largely adult composition of the sample may affect how neglect is perceived and reported, since neglect tends to have greater relevance in the context of minors who rely on others for care and protection.

While these findings might initially suggest that sports are not inherently more prone to IV than other life contexts (e.g., school, family, peer groups), this interpretation requires caution. The present study did not assess non-sports contexts separately but compared sports to a combined reference category. As such, our findings reflect an aggregated contrast rather than a differentiated analysis of risk across specific domains. Moreover, exposure time, relationship structures, and situational dynamics differ substantially between environments such as schools and sports clubs. Given the relatively limited time many individuals spend in sports settings, the consistently high prevalence of IV reported in this context remains noteworthy.

Importantly, the substantial overlap in IV experiences across contexts suggests that sports environments mirror broader societal dynamics. This raises critical questions about the role sports clubs may play—either in perpetuating existing patterns of abuse or serving as protective and preventive spaces. Research from Canadian youth athlete samples has shown that child maltreatment outside sports is associated with increased risk of IV within sports (35). These findings support the idea that prior victimization may increase vulnerability to revictimization, reinforcing the need to understand IV in sports within a broader life-course framework (16, 60).

### 4.3 Gender

In our sample, women reported significantly more IV inside sports than men (77.1% vs. 63.6%). This difference was particularly pronounced for sexual violence: both non-contact and contact forms were over twice as likely to be reported by women, a pattern confirmed in multiple regression models (OR > 2). These findings are consistent with a broad body of research highlighting the heightened risk of sexual violence for female athletes, often linked to gendered power dynamics in sports (12, 13, 32, 33, 61). However, findings from Hartill et al. (16) offer a contrasting perspective: in their multi-country European sample, boys reported higher prevalence across all forms of IV, including sexual violence, with these differences diminishing when adjusting for other influences. Although the study by Hartill et al. (16) presents differing findings, it remains the only investigation to date reporting higher rates of sexual violence among male athletes. One possible explanation is that Hartill et al. surveyed a comparatively young cohort (18–30 years, mean age 24.4 years). Conversely, our study assessed lifetime prevalence in a sample with an average age above 40. It is conceivable that among younger generations, men may feel less constrained by taboos surrounding the disclosure of sexual violence and may be more willing to recognize and report such experiences, particularly in the context of an anonymous survey. In older cohorts, however, sexual violence against men may have been more strongly normalized or silenced, potentially contributing to lower reported rates. Given this and considering the methodological limitations of their recruitment strategy, our findings should not be interpreted as an outlier but rather as consistent with a broader evidence base suggesting that women are more frequently affected. Nonetheless, our data also underscore that boys and men are affected by sexual violence in substantial numbers—highlighting the importance of ensuring that prevention and support strategies address the needs of all genders.

For physical violence, our descriptive results showed a slightly higher prevalence among women (39.1% vs. 34.9%), yet men had a higher adjusted risk in multivariate models after controlling for other factors. This pattern is consistent with several studies reporting higher rates of physical violence among boys and men, particularly in team sports, where aggressive behavior may be more normalized or reinforced through sport-specific culture (16, 24, 62).

No significant gender differences were found for psychological violence or neglect in our regression models, although women reported slightly higher prevalence in descriptive terms. While some studies similarly report no clear gender effects for these forms of violence (12, 16, 63), other research has found that women may be more frequently exposed to psychological violence and neglect (13, 24, 54). This suggests that gendered dynamics may not always manifest in overall prevalence rates but can still shape how psychological violence and neglect are experienced, tolerated, or reported in sports.

Taken together, these findings underline the complexity of gendered risk in sport. Meta-analyses of available IV prevalence reports would help clarify patterns and inform more targeted prevention. While women are clearly more affected by sexual violence in our sample, male athletes are not exempt from IV, particularly in the form of physical violence. Prevention strategies should therefore avoid simplistic gender assumptions and instead account for the diverse, form-specific, and intersecting risks faced by all athletes.

### 4.4 Age

Age-related patterns in IV remain underexamined in broader club or adult samples, as most research to date has focused on youth sport. Our findings help address this gap by including a wide age range of athletes. It is important to note that age in our study mostly referred to participants' current age-group, not their age at the time of the incident. One exception was made by contextualizing our core findings with data about the subjectively most severe incident for each form of IV. Those findings indicate that a substantial proportion of IV experiences in sport begin or continue after the age of 18, thus challenging the assumption that IV in sport is primarily a youth phenomenon.

While descriptive analyses indicated that older participants (aged 41 and above) generally reported lower overall IV prevalence than younger participants—especially those aged 16–18—multivariate models revealed a more differentiated pattern: Older participants reported significantly fewer incidents of physical violence and neglect. In contrast, for NCSV, older age groups in our study showed a higher likelihood of reporting such experiences. Our results for psychological violence and CSV, showed no significant age-related differences. The IV sub forms physical violence and neglect might be more salient during youth, when individuals are more dependent on caregivers (or coaches), and require age adequate organizational structures (7). The lower prevalence reported by older participants for those two forms may also reflect recall bias, or a tendency to reinterpret earlier experiences as less severe over time, especially given that perceptions of what constitutes harmful or abusive behavior may have changed across time. Given that our data do not capture the timing of every IV incident, no causal conclusions can be drawn about the actual risk of IV for each age groups. Comparable literature on associations between age and IV risk is scarce and results are mixed. For instance, Parent & Vaillancourt-Morel (24) questioned a sample of adolescents (aged 14–17) and found that older adolescents reported more psychological and physical violence, and neglect than younger ones, which they attributed to greater exposure time. Vertommen et al. (11) reported no differences in physical or sexual violence across younger and older age cohorts, while psychological violence was significantly lower among older respondents. The difference from our findings—and those of Vertommen et al. (11) - likely stems from the narrower age range in Parent & Vaillancourt-Morel's sample (24), as well as their focus on adolescence, whereas both our study and Vertommen's included adult participants. Thus, observed age effects may vary depending on the life stage assessed and the cumulative nature of exposure. Methodological differences may also account for divergent findings. Our study distinguished between contact and non-contact sexual violence, while Vertommen et al. (10) analyzed sexual violence as a single aggregated measure, potentially masking form-specific patterns.

Overall, while age associations varied by IV form, the evidence that some incidents might begin or persist after the age of 18 underscores that IV is not solely a youth issue in sport. Safeguarding efforts should therefore be age-sensitive and extend beyond youth-focused frameworks. Future research could further explore how age, exposure time, and changing social norms shape the reporting and interpretation of IV experiences.

### 4.5 Level of sports, weekly training time and early specialization

German sports clubs offer a wide variety of sports activities, ranging from recreational, and non-competitive sports to high-level elite sports. This diversity in athletic ambition is well represented in the survey sample. Sports club members who were active only in recreational, non-competitive sports generally reported lower rated of IV inside sports than those pursuing competitive sports. Our results indicate a tendency toward higher IV prevalence at elevated competition levels, which corresponds with findings by Ohlert et al. (12), who reported a higher prevalence of IV among elite athletes compared to the overall prevalence observed in our study. These findings are also consistent with Vertommen et al. (11), who found that former international-level youth athletes reported significantly higher exposure to IV, in line with elevated risk profiles observed within elite-level sport.

In the case of physical violence, being an international-level athlete was associated with higher odds of reporting IV, potentially reflecting a mentality of competing and training “at all costs”. A similar pattern emerged among athletes who had specialized early in a single sport before the age of 12. These participants reported physical violence at significantly higher rates, supporting previous research that has linked early specialization to increased vulnerability to IV: Parent & Vaillancourt-Morel (24) found that early sports specialization and increased training hours were associated with higher risks of psychological violence and neglect. They interpret these risks in relation to overdependence, pressure, and institutional dynamics that may normalize harmful training environments.

As the competition level increases, the time spent in training or competition usually also increases. The amount of time spent in training or within the sports environment (such as in more intense club program), is thus likely a confounding factor for the higher prevalence in higher-level sports and was evaluated alongside the athletic level. In our findings, similar to athletic level, the risk for psychological and physical violence, as well as neglect is more likely to increase as training time increases, which aligns with previous work highlighting the link between intensive training loads and increased exposure to IV (24). However, no such trend was found for either form of sexual violence.

Although the influence of athletic level and training time diminished in the multiple regression models compared to broader predictors such as victimization outside of sports or multi-victimization within sport, these sport-specific variables remain relevant. They should be investigated further to better identify risk profiles, particularly for athletes in performance-oriented sports environments.

Although our cross-sectional data only establish associations, there is indication that performance-oriented environments may be connected to a higher risk of IV in sport (5) Performance pressure, higher-level competition, and “winning-centric” cultures may normalize harmful behaviors. However, IV can also occur in less performance-driven contexts such as recreational sport and its risk should not be attributed to performance orientation alone. Accordingly, IV risk is increasingly understood as the result of a complex interaction between performance norms, organizational climate, hierarchical structures, and broader social dynamics, as posited by Bronfenbrenner's ecological systems theory (5, 36).

### 4.6 Overlap in forms of IV

The co-occurrence of several forms of IV characterizes many cases of violence in sport, reinforcing that focusing solely on a single form—such as sexual violence—would be insufficient for effective prevention. Psychological violence frequently accompanies other forms of IV, suggesting a potentially enabling or gateway function that lowers boundaries and may normalize harmful dynamics. This notion would support the importance of early intervention in response to psychological boundary violations.

Although targeted research and prevention efforts focusing on sexual violence remain important—particularly given its stigma and underreporting—IV forms must be understood as interconnected. Our findings align with prior research showing that different forms of IV frequently overlap rather than occur in isolation (11, 12, 16). This pattern of poly-victimization is linked to more severe long-term outcomes, such as increased psychological distress and reduced quality of life (40). Notably, similar co-occurrence patterns have been documented outside of sport—particularly in childhood adversity and family violence research—where multiple forms of abuse often cluster and compound harm (29, 31).

In line with international consensus, including the IOC safeguarding framework (5), our results support the need for comprehensive, athlete-centered prevention strategies that recognize the cumulative and syndromic nature of violence in sport. Preventive frameworks must therefore address the full spectrum of IV rather than treating each type in isolation.

### 4.7 Limitations

While the questionnaire used in this study was comprehensive and closely aligned with established instruments assessing IV in sports, it has not undergone formal psychometric validation. Nevertheless, the structure and content were based on validated tools such as IVACS-Q (16) and IVIS (11, 12), enabling content validity and a high degree of methodological comparability within the sports context. A key limitation, however, lies in the limited comparability with validated instruments for assessing Adverse Childhood Experiences (ACEs), which are often used in broader public health research. Since IV cannot be fully understood in isolation from other life domains, future measurement tools should strive to bridge this gap by integrating sports-specific and general life-context adversities. This would allow for a more holistic assessment of lifetime exposure to IV—especially where experiences in and outside of sports may overlap or compound one another.

Due to the cross-sectional design of this survey, no inferences can be made in terms of chronology or causation. Data are not triangulated with administrative records or observational data. As a retrospective self-report measure was used, the results might also be inherently biased, e.g., through non-response bias, as individuals who chose not to participate may differ systematically from those who did. In addition, the digital survey format could have had a limiting effect regarding participation of individuals with lower access to or familiarity with digital technologies. Although the survey was conducted anonymously, the sensitive nature of some topics may have led to mild social desirability bias, potentially resulting in underreporting of certain experiences, especially in performance-oriented sport environments where loyalty to coaches and teams, fear of negative career consequences, and normalization of harsh practices may discourage full disclosure. In the same manner, memory bias, trauma-related recall difficulties, or avoidance might have affected retrospective self-reports, as shown in research on abuse and interpersonal violence in sport, where delayed disclosure and fragmented recall are common. Taken together, these factors likely bias prevalence estimates of interpersonal violence in sport downwards rather than upwards, suggesting that the true burden may be higher than reported.

Women seem to be slightly overrepresented in the sample, whereas the age distribution appears to correspond to the available demographic data on sports club members (50). However, no conclusions can be drawn regarding the representativeness of the sample due to limited data and the use of a self-selected convenience sample with a relatively low response rate. The results are therefore interpreted as insights into patterns and associations within this accessible segment.

Less than 5% of respondents in our sample were former sports club members. As a result, the experiences of individuals who have discontinued participation in organized sports are underrepresented. This is particularly relevant given the possibility that IV experiences may have contributed to sports dropout. If interpersonal violence contributed to discontinuation, those most affected may be missing from our data, potentially leading to an underestimation of both prevalence and severity of IV in sport. Our recruitment strategy—primarily through sports federations and affiliated networks—relied on sport-specific communication channels and likely favored current members. Such a strategy inherently limits access to individuals who have disengaged from club sports, potentially excluding those with more negative or disruptive experiences. Future research should aim to include this group more systematically, as their perspectives may differ in important ways. One potential solution could be the integration of sport-related IV items into population-based surveys, allowing for more representative sampling that includes those no longer active in sport.

### 4.8 Conclusion

This study is the first to highlight the issue of IV in a sample of adult sports club members in Germany. In addition to the reported high prevalence of IV against athletes in German sports clubs, three findings are particularly striking. First, the results showed that IV is not merely a problem in elite or youth sports but occurs on all levels including adult recreational sports. Yet, specific factors, such as individual or sport-club specific traits, or sports-specific characteristics, such as a higher level or time spent in training, need to be investigated more closely to differentiate the risk for certain sub-groups. Those findings could be used to tailor evidence-based prevention and intervention programs that should not only focus on minors but also on adults, because protection of children will only work in an environment or organization where all participants feel safe.

Second, different forms of IV often co-occur, especially in conjunction with psychological violence. While our cross-sectional data do not allow temporal conclusion, qualitative reports indicate that psychological violence precedes other forms of IV and is often used as a means to control by continually overstepping personal boundaries, in preparation for and concurrently with other forms of IV (59). Therefore, it is sensible to consider all forms of IV, and especially psychological violence, in preventive efforts. It should be the task of every sports club to delineate acceptable and unacceptable behavior to eradicate “grey areas” in their sports practice, and intervene when any transgression is suspected, as more profound issues might underlie.

Third, experiences within and outside sports contexts often overlap. The high frequency of IV inside sports is thus hardly surprising in relation to contexts other than sports. The prevention of IV in sports should be seen as a task for society, as the causes of IV in sports cannot be attributed exclusively to the particularities of the sports system. Measures to prevent IV in sports must be coordinated with measures outside sports to create synergies and to avoid loss of resources. At the same time, sports clubs, as central providers of recreational activities, hold a special responsibility. Many of those affected by IV are members of sports clubs and experience IV in such contexts either for the first time or repeatedly in what should be a safe and beneficial environment. Education-based interventions for all sports club stakeholders need to be developed and implemented, and support for club administrators to establish safeguarding procedures should be provided. By empowering sports clubs to recognize their dual role – potential sites of abuse and potential sources of support – clubs can be equipped to prevent re-victimization and offer resources to those already affected. Knowledge and prevention of (re-)victimization should thus play a larger role in the prevention of IV, as sports clubs could ideally be places where those affected might receive support. Against the backdrop of these findings, it thus seems important to view sports clubs as possible scenes of IV on the one hand but also as places where those who have already experienced IV outside of sports could be able to find empowering resources, provided they are not re-victimized.

## Data Availability

The raw data supporting the conclusions of this article will be made available by the authors, without undue reservation.
