# Supplementary material for: Prevalence of interpersonal violence in sports clubs in Germany
Source: Front Sports Act Living. 2026 Feb 26;8:1701609. doi: 10.3389/fspor.2026.1701609 (PMC12979139; doi:10.3389/fspor.2026.1701609)
Supplement: Supplementary file 1 [file Datasheet1.pdf]

Supplementary Material 1

German questionnaire items on interpersonal violence in sports

After each item, the source of the item is mentioned, including item number and form of IV.

- IVACS: Interpersonal Violence Against Children in Sport Questionnaire; Hartill M, Rulofs B, Allroggen M, Demarbaix S, Diketmüller R, Lang M, Martin M, Nanu I, Sage D, Stativa E, et al. Prevalence of interpersonal violence against children in sport in six European countries. *Child Abuse & Neglect* (2023) 146:106513. doi: 10.1016/j.chiabu.2023.106513
- IVIS: Interpersonal violence in sport questionnaire; Vertommen T, Schipper-van Veldhoven N, Wouters K, Kampen JK, Brackenridge CH, Rhind DJA, Neels K, Van Den Eede F. Interpersonal violence against children in sport in the Netherlands and Belgium. *Child Abuse & Neglect* (2016) 51:223–236.
- Kerr et al. (2019): Kerr G, Willson E, Kin B, Stirling A. Athletes CAN: Prevalence of Maltreatment Among Current and Former National Team Athletes. (2019)51.
- "adapted from" indicates that the item differs slightly from the original question.

The English translation *(in brackets and italics)* is provided for clarity only. The questionnaire has not been professionally translated into English.

Skala *(response scale)*

- Dies ist mir nie im Sport passiert (1*) (this has never happened to me in sport)*
- Dies ist mir einmal im Sport passiert (2*) (this has happened to me once in sport)*
- Dies ist mir zwei- bis viermal im Sport passiert (3) *(this has happened to me two to four times in sport)*
- Dies ist mir fünfmal oder öfter im Sport passiert *(4) (this has happened to me five times or more in sport)*

Psychische Gewalt *(Psychological violence)*

Manchmal können Menschen Dinge tun oder sagen, die die Gefühle von anderen Menschen verletzen oder negative emotionale Auswirkungen haben.

Wie häufig haben Sie bisher in Ihrem Leben die folgenden Situationen im Vereinssport oder im Umfeld des Vereinssports erlebt – egal durch welche Person/en?

Es kann sich auch um Situationen handeln, die Sie erst im Nachhinein als unangemessen erlebt haben.

Sometimes people may do or say things that hurt the feelings of others or have negative emotional effects. How often in your life have you experienced the following situations in club sport or in the context of club sport – regardless of who the person/people were? These may also include situations you only recognized as inappropriate later on.

EG1 Sie wurden runtergemacht, in große Verlegenheit gebracht oder gedemütigt, z.B. durch abwertende Spitznamen *(IVACS PSYV7, IVIS PSYV3)*

(You were put down, embarrassed, or humiliated, e.g., through derogatory nicknames.)

EG2 Sie wurden wegen Ihrer körperlichen Erscheinung kritisiert, z.B. wegen Ihres/r Gewichts, Aussehens, Kleidung, Körperform *(IVACS PSYV8, IVIS PSYV5)*

(*You were criticized for your physical appearance, e.g., weight, looks, clothing, or body shape.)*

EG3 Sie wurden wegen Ihrer Leistung im Wettkampf oder Training persönlich angegriffen, beschimpft oder bedroht *(adapted from IVACS PSYV 11 and IVIS PSYV6)*

(You were personally attacked, insulted, or threatened because of your performance in competition or training.)

EG4 Sie wurden persönlich angegriffen, beschimpft oder bedroht, weil Sie nicht an einem Training oder Wettkampf teilnehmen wollten *(adapted from IVACS PSYV14 and IVIS PSYV9)*

(*You were personally attacked, insulted, or threatened because you did not want to participate in training or competition.)*

EG5 Sie wurden grundlos und absichtlich ignoriert oder ausgeschlossen *(adapted from IVACS PSYV9 and IVIS PSYV8)*

(*You were deliberately ignored or excluded without cause.)*

EG6 Menschen aus dem Verein oder dem Umfeld des Sports haben schlecht über Sie geredet oder Lügen über Sie erzählt (*IVIS PSYV10)*

(People at the club or in the sports environment talked badly about you or spread lies about you.)

EG7 Um Sie zu erniedrigen oder Ihnen Angst zu machen, wurden Ihre Sachen beschädigt oder gestohlen *(IVIS PSYV11)*

(*Your belongings were damaged or stolen to intimidate or humiliate you.)*

EG8 Sie wurden aus Gründen, die nichts mit Ihrer Leistung oder Ihrem Verhalten zu tun hatten, oder aus nicht erläuterten Gründen, aus Ihrem Team/Verein/Gruppe ausgeschlossen, oder Ihnen wurde damit gedroht *(IVACS PSYV 15, IVIS PSYV12)*

(You were excluded or threatened with exclusion from your team/club for reasons unrelated to your performance or behaviour, or without explanation.)

EG9 Ihnen wurde körperliche Gewalt angedroht, ohne dass Sie tatsächlich angegriffen wurden *(adapted from IVIS PSYV13)*

(You were threatened with physical violence without being actually attacked.)

EG10 Sie wurden aufgefordert oder gezwungen, an Aufnahme- oder anderen Ritualen teilzunehmen, die damit verbunden waren, Sie (oder andere) zu demütigen, zu erniedrigen oder herabzuwürdigen *(IVACS PSYV13).*

(You were instructed or forced to participate in initiation ceremonies or other rituals intended to humiliate, degrade or belittle yourself or others.)

EG11 Sie wurden aufgefordert, angewiesen oder gezwungen, unrealistisch hohe Erwartungen zu erfüllen. (INFO BUTTON: „unrealistisch hohe Erwartungen" für Ihr Alter, Ihre Gesundheit, Ihr Leistungsvermögen, Ihre Fitness) *(IVACS PSYV12)*

(You were asked, instructed, or forced to meet unrealistically high expectations. INFO BUTTON: "unrealistic expectation" for your age, your health, you abilities or your fitness.)

EG12 Sie wurden für besondere Bemühungen oder Leistungen nicht gelobt oder sogar ignoriert *(IVACS PSYV10)*

(You were not praised for special efforts or achievements or were even ignored.)

EG13 Sie hatten das Gefühl, dass einige Sportler*innen anderen Sportler*innen vorgezogen wurden *(adapted from Kerr et al. (2019)).*

(You felt that some athletes were favoured over others.)

Körperliche Gewalt *(physical violence)*

Manchmal können Menschen Dinge tun, die andere körperlich verletzen.

Wie häufig haben Sie bisher in Ihrem Leben die hier aufgelisteten Situationen im Vereinssport oder im Umfeld des Sports erlebt – egal durch welche Person(en)?

Es kann sich auch um Situationen handeln, die Sie erst im Nachhinein als unangemessen erlebt haben.

Wichtig: Hier geht es NICHT um Situationen und Techniken, die Bestandteil Ihrer Sportausübung sind, also z.B. nicht um Kampf- und Schlagtechniken, Bodychecks beim Eishockey, Tackling beim Rugby oder ähnliches.

Sometimes people may do things that physically harm others. How often in your life have you experienced the following situations in club sport or in the context of sport – regardless of who the person/people were? These may include situations you only recognized as inappropriate later. Important: This does NOT refer to techniques that are part of your sport, e.g. fighting or hitting techniques, bodychecks during ice hockey, tackling during rugby or similar.

KG1 Sie wurden geschüttelt *(IVIS PHV2)*

(You were shaken.)

KG2 Es wurden Dinge nach Ihnen geworfen *(IVIS PHV 3)*

(Objects were thrown at you.)

KG3 Sie wurden festgehalten oder mit Gewalt zurückgehalten *(IVIS PHV 4)*

(*You were held or physically restrained.)*

KG4 Sie wurden mit der offenen Hand geschlagen *(IVIS PHV 5)*

(*You were slapped with an open hand.)*

KG5 Sie wurden mit der Faust geschlagen oder geboxt *(IVIS PHV 6)*

(You were punched with a fist.)

KG6 Sie wurden mit einem Gegenstand geschlagen (z. B. einem Schuh oder Schläger) *(IVIS PHV 7)*

(*You were hit with an object (e.g., shoe, stick).)*

KG7 Sie wurden auf den Boden gezwungen oder umgerissen *(IVIS PHV 8)*

(*You were forced to the ground or knocked over.)*

KG8 Sie wurden am Hals gepackt oder gewürgt *(IVIS PHV 9)*

(*You were grabbed by the neck or choked.)*

KG9 Sie wurden verprügelt *(IVIS PHV 10)*

(You were beaten up.)

KG10 Sie wurden dazu gezwungen zu trainieren oder an einem Wettkampf teilzunehmen, während Sie verletzt, krank oder ausgelaugt waren oder in einer Intensität oder Häufigkeit zu trainieren oder an Wettkämpfen teilzunehmen, die möglicherweise schädlich war *(IVACS PHV19, IVIS PHV1)*

(You were forced to train or compete while injured, ill, exhausted or at a harmful intensity/frequency.)

KG11 Sie wurden dazu gezwungen Sport als eine Form der Bestrafung auszuüben (z.B. Liegestütze oder Extrarunden) *(IVACS PHV16)*

(*You were forced to perform sport as a form of punishment (e.g. push-ups or extra rounds.).*

KG12 Sie wurden dazu gezwungen an Aufnahme- oder anderen Ritualen, die verletzende körperliche Aktivitäten beinhalteten, teilzunehmen (z.B. Schläge, Strangulierung, übermäßiger Alkoholkonsum) *(IVACS PHV17)*

(You were forced to participate in ceremonies or other rituals involving harmful physical activities, e.g. hitting, choking, excessive alcohol consumption.)

KG13 Sie wurden dazu gezwungen Substanzen zu sich zu nehmen, um Ihr Körpergewicht zu regulieren (INFO BUTTON: um Gewicht zu verlieren oder Muskelmasse aufzubauen), Ihre Leistungsfähigkeit zu steigern, den Beginn Ihrer Pubertät zu verzögern oder Ihre Menstruation zu unterbinden *(IVACS PHV18, IVIS PHV 14)*

(You were forced to take substances to control weight (INFO BUTTON:to lose weight or to gain muscle mass), enhance performance, delay puberty, or suppress menstruation.)

Information: IVACS combines items KG1–KG9 into the following single item:

I was punched, slapped, grabbed / pushed, or otherwise physically assaulted. (IVACS PHV20).

Sexualisierte Gewalt ohne Körperkontakt *(non-contact sexual violence NCSV)*

Wie häufig haben Sie bisher in Ihrem Leben die folgenden Situationen im Vereinssport oder im Umfeld des Sports erlebt – egal durch welche Person/-en?

Es kann sich auch um Situationen handeln, die Sie erst im Nachhinein als unangemessen erlebt haben.

(How often have you experienced the following situations in club sport or the context of sport – regardless of who the person/people were? This may also include situations you only later recognized as inappropriate.)

SGO1 Sie waren anzüglichen oder sexuellen Kommentaren ausgesetzt. Zum Beispiel sexuelle Witze, Bemerkungen über Ihren Körper, Beleidigungen zu Ihrer Männlichkeit oder Weiblichkeit *(adapted from IVACS NCSV21 and IVIS SV1)*

(*You were exposed to lewd or sexual comments, e.g. sexualized jokes, comments about your body, insults towards your masculinity or femineity.)*

SGO2 Ihnen wurde nachgepfiffen oder in sexuell anzüglicher Weise nachgerufen *(IVIS SV2)*

(*You were whistled at or called at in a sexually suggestive manner.)*

SGO3 Über Ihren Körper und Ihr Aussehen wurden sexuell anzügliche Bemerkungen gemacht *(IVIS SV3)*

(Sexually suggestive comments were made about your body or appearance.)

SGO4 Sie wurden mit sexuell anzüglichen Blicken angesehen oder angestarrt *(adapted from IVACS NCSV22 and IVIS SV4)*

(You were stared at or leered at with sexually suggestive looks.)

SGO5 Sie wurden gebeten, mit jemandem alleine zu sein, was für Sie unangenehm war *(IVIS SV9)*

(You were asked to be alone with someone in a situation that made you uncomfortable.)

SGO6 Jemand hat sich gegen Ihren Willen vor Ihnen persönlich oder über soziale Medien vor Ihnen entblößt (z.B. Genitalien, Brüste, Gesäß) *(adapted from IVACS NCSV27&28, IVIS SV14).*

Someone exposed themselves to you against your will in person or online (e.g. genitals, breast or buttocks).

SGO7 Sie haben ungewollt Anrufe, Zettel, Mails, Texte, Fotos oder Videos (gezeigt) bekommen (vielleicht über Ihr Handy oder das Internet), die sexuelle Andeutungen enthielten oder eindeutig sexuell waren *(IVIS SV10)*

(*You have received unsolicited calls, notes, emails, texts, photos or videos (perhaps via your mobile phone or the internet) that contained sexual innuendo or were explicitly sexual.)*

SGO8 Sie wurden aufgefordert, angewiesen oder gezwungen, Bilder, Videos oder Nachrichten mit sexuellem Inhalt anzusehen *(IVACS NCSV23)*

(*You were asked, instructed, or forced to view images, videos, or messages with sexual content.)*

SGO9 Sexuelle Bilder von Ihnen wurden von jemand anderem aufgenommen oder geteilt *(IVACS NCSV25, IVIS SV25)*

(Sexual images of you were taken or shared by someone else.)

SGO10 Sie wurden aufgefordert, angewiesen oder gezwungen, Bilder, Videos oder Textnachrichten mit sexuellem Inhalt von sich oder anderen Personen zu erstellen oder zu teilen *(IVACS NCSV 24)*

(You were asked, instructed, or forced to create or share sexually explicit images, videos, or text messages of themselves or others.)

SGO11 Sie wurden aufgefordert, angewiesen oder gezwungen, sich auszuziehen oder sexuelle Handlungen an sich selbst zum Vergnügen einer anderen Person/anderer Personen durchzuführen *(IVACS NCSV26)*

(You were asked, instructed, or forced to undress or perform sexual acts on yourself for the pleasure of another person/people.)

SGO12 Jemand wollte, dass Sie sich ausziehen, sich eindeutig sexuell darstellen oder Geschlechtsverkehr haben, während jemand anderes anwesend war (mit oder ohne Kamera) oder während jemand über das Internet zugeschaut hat *(IVIS SV13)*

(*Someone wanted you to undress, perform in a clearly sexual manner, or have sexual intercourse while someone else was present (with or without a camera) or while someone was watching via the internet.)*

SGO13 Sie wurden aufgefordert oder gezwungen, an Aufnahme- oder anderen Ritualen teilzunehmen, einschließlich erniedrigender oder verletzender Aktivitäten sexueller Art, ohne Körperkontakt *(IVACS NCSV29)*

(Your were asked or forced to participate in initiation or other rituals, including degrading or abusive sexual activities, without physical contact.)

SGO14 Von Ihnen wurden Fotos gemacht, die Ihre Behinderung betont haben, so dass Sie das Gefühl hatten, es hat die Person sexuell erregt oder Sie sollten Ihre Behinderung explizit vorführen. *[nur wenn Behinderung – extra Frage]*

(Photos were taken of you that emphasized your disability, leading you to feel that it sexually aroused the person or that you were expected to explicitly display your disability. [only if disability – extra question])

Sexualisierte Gewalt mit Körperkontakt *(sexual violence involving physical contact)*

Nachdem wir Sie gerade zu möglicherweise von Ihnen gemachten Erfahrungen sexueller Art ohne Körperkontakt befragt haben, möchten wir Sie bitten, im Folgenden weitere Fragen zu beantworten. Diese werden sich mit von Ihnen möglicherweise im Vereinssport gemachten Erfahrungen sexueller Art mit Körperkontakt befassen.

Wie häufig haben Sie bisher in Ihrem Leben die folgenden Situationen im Vereinssport oder im Umfeld des Sports erlebt – egal durch welche Person/-en?

(Having just asked you about any sexual experiences you may have had without physical contact, we would now like to ask you to answer the following questions. These will address any sexual experiences you may have had involving physical contact in club sports. How often in your life have you experienced the following situations in club sports or in the sports environment – regardless of the person(s) involved?)

SGK1 Jemand kam so nah zu Ihnen, dass es für Sie unangenehm war *(IVIS SV5)*

(Someone came so close to you that it was uncomfortable for you.)

SGK2 Es gab körperlichen Kontakt, der für Sie unangenehm war (z.B. jemand lehnte sich an Sie an) *(IVIS SV6)*

(There was physical contact that was unpleasant for you (e.g., someone leaned against you).)

SGK3 Sie wurden beim Training in einer Weise berührt, die für Sie unangenehm war *(IVIS SV7)*

(You were touched in a way during training that was unpleasant for you.)

SGK4 Sie wurden auf eine Weise eingerieben oder massiert, die für Sie unangenehm war *(IVIS SV8)*

(You were rubbed or massaged in a way that was unpleasant for you.)

SGK5 Sie wurden dazu gedrängt oder gezwungen, jemanden gegen Ihren Willen zu küssen oder wurden gegen Ihren Willen von jemandem geküsst *(adapted from IVACS CSV30, IVIS SV12)*

(*You were pressured or forced to kiss someone against your will, or you were kissed against your will.)*

SGK6 Sie wurden gegen Ihren Willen gestreichelt oder anderweitig sexuell berührt *(adapted from IVACS CSV31 and IVIS SV15)*

(You were stroked or otherwise sexually touched against your will.)

SGK7 Sie wurden aufgefordert, angewiesen oder gezwungen, jemanden sexuell zu berühren *(adapted from IVACS CSV31, split up into two questions).*

(You were asked, instructed, or forced to sexually touch someone.)

SGK8 Jemand hat versucht, gegen Ihren Willen Sex mit Ihnen zu haben *(IVIS SV16)*

(Someone tried to have sex with you against your will.)

SGK9 Sie wurden zu Oralsex gezwungen (von Ihnen durchgeführt oder an Ihnen durchgeführt) *(adapted from IVACS CSV 33 and IVIS SV17).*

(You were forced to perform oral sex (either by you or on you).)

SGK10 Sie wurden zu vaginalem oder analem Sex gezwungen (mit einem Objekt oder einer Person) *(adapted from IVACS CSV 34 and IVIS SV17)*

(*You were forced to have vaginal or anal sex (with an object or a person).)*

SGK11 Sie wurden aufgefordert oder gezwungen, an Aufnahme- oder anderen Ritualen teilzunehmen, einschließlich erniedrigender oder verletzender Aktivitäten sexueller Art, die Körperkontakt beinhalteten *(IVACS CSV35)*

(*You were asked or forced to participate in initiation or other rituals, including degrading or abusive sexual activities involving physical contact.)*

Vernachlässigung

Manchmal kümmern sich die Menschen, die für Sportler*innen Verantwortung tragen, nicht so um sie, wie sie es sollten.

Wie häufig haben Sie bisher in Ihrem Leben die folgenden Situationen im Vereinssport oder im Umfeld des Sports erlebt – egal durch welche Person/-en?

(Sometimes, the people responsible for athletes don't take care of them as well as they should. How often have you experienced the following situations in your life, either in club sports or in the sports community – regardless of the person(s) involved?)

NE1 Sie erhielten keine ausreichende Versorgung für die Befriedigung körperlicher Grundbedürfnisse, z.B. nicht genug Essen/ Trinken/ Schlaf, nicht auf die Toilette gehen dürfen. *(adapted from IVACS NE1)*

(*You did not receive sufficient provision to satisfy basic physical needs, e.g. not enough food/drink/sleep, not being allowed to go to the toilet.)*

NE2 Sie haben trotz Bedarf (z.B. Verletzung) keine angemessene medizinische Versorgung erhalten. *(adapted from IVACS NE2)*

(*Despite needing it (e.g., due to injury), you did not receive adequate medical care.)*

NE3 Sie wurden ohne ausreichende Beaufsichtigung, Fürsorge oder Betreuung alleine gelassen, sodass Sie dem Risiko einer Verletzung ausgesetzt waren. *(adapted from IVACS NE3)*

(You were left alone without adequate supervision, care or support, leaving you at risk of injury.)

NE4 Ihnen wurde nicht die geeignete Ausrüstung zur Verfügung gestellt, um Ihre sportlichen Aktivitäten sicher auszuführen (z.B. zugelassene Ausrüstung, Ausrüstung in gutem Zustand). *(adapted from IVACS NE4)*

(You were not provided with the appropriate equipment to safely carry out your sporting activities (e.g., approved equipment, equipment in good condition).)

NE5 Sie wurden angewiesen oder gezwungen, in der Schule/Ausbildung/Hochschule/im Beruf zu fehlen, um sportlichen Aktivitäten oder Aufgaben im Verein nachzugehen. *(adapted from IVACS NE5)*

(You were instructed or forced to miss school/training/university/work to participate in sports activities or club duties.)

NE6 Sie wurden gezwungen unter unsicheren Bedingungen an Trainings oder Wettkämpfen teilzunehmen, z.B. unter extremen Wetterbedingungen, auf unsicheren Sportanlagen oder unter Missachtung von Sicherheitsregeln. *(adapted from IVACS NE6)*

(You were forced to participate in training or competitions under unsafe conditions, e.g., in extreme weather conditions, on unsafe sports facilities, or in disregard of safety rules.)

NE7 Wenn Sie vor (neuen) Trainingsinhalten Angst oder Sicherheitsbedenken geäußert haben, wurden Ihre Einwände ignoriert oder heruntergespielt. *(adapted from Kerr et al. 2019)*

(*If you expressed fear or safety concerns regarding (new) training content, your objections were ignored or downplayed.)*
