# Supplementary material for: Prevalence of interpersonal violence in sports clubs in Germany
Source: Front Sports Act Living. 2026 Feb 26;8:1701609. doi: 10.3389/fspor.2026.1701609 (PMC12979139; doi:10.3389/fspor.2026.1701609)
Supplement: Supplementary file 2 [file Datasheet2.pdf]

Supplementary Material 2

Supplementary Table

Simple and multiple regression results of gender, age, athletic level, training time per week, early specialization, IV outside of sports, and forms of IV inside sports for each sub-form of IV.

**Psychological violence**		Simple logistic regression				Multiple logistic regression			
$R^2_{Nagelkerke}$=.499	Indicator	OR	99% CI		p	OR	99% CI		p
(multiple log reg)			Lower	Upper			Lower	Upper	
Gender	Male[O]								
	Female	1.692	1.430	2.002	<.001	1.072	.815	1.410	.515
Age	16 to 18 years[O]								
	19 to 26 years	1.471	1.010	2.141	.008	1.412	.843	2.362	.085
	27 to 40 years	1.083	.751	1.562	.572	1.194	.724	1.971	.362
	41 to 60 years	.472	.338	.658	<.001	.891	.558	1.423	.525
	61 years	.254	.177	.365	<.001	.833	.498	1.395	.362
Athletic level	Recreational[O]								
	Local	1.907	1.495	2.432	<.001	1.484	1.056	2.086	.003
	Regional	3.094	2.386	4.013	<.001	2.078	1.437	3.005	<.001
	National	2.931	2.160	3.976	<.001	1.676	1.072	2.619	.003
	International	3.983	2.588	6.131	<.001	2.010	1.073	3.765	.004

**Psychological violence**		Simple logistic regression				Multiple logistic regression			
Training time/week	< 2 hours[O]								
	2.5 - 6 hours	3.326	1.592	2.390	<.001	1.388	1.046	1.842	.003
	6.5 - 10 hours	4.573	2.511	4.407	<.001	1.696	1.137	2.529	<.001
	10.5 - 14 hours	5.908	2.771	7.546	<.001	2.843	1.384	5.838	<.001
	14.5 - 20 hours	4.007	2.664	13.103	<.001	2.422	.866	6.776	.027
	> 20 hours	1.951	1.572	10.215	<.001	1.484	.423	5.201	.418
Early specialization	No[O]								
	Yes	2.093	1.630	2.687	<.001	1.194	.846	1.684	.185
IV outside sports	Psych. violence	9.733	8.042	11.778	<.001	6.468	4.994	8.378	<.001
	Physical violence	2.832	2.348	3.417	<.001	.815	.611	1.088	.068
	NCSV	2.843	2.342	3.450	<.001	.804	.560	1.154	.119
	CSV	2.299	1.892	2.794	<.001	.882	.621	1.254	.360
	Neglect	4.013	2.685	5.999	<.001	1.044	.588	1.853	.847
IV inside sports	Physical violence	7.528	6.011	9.427	<.001	2.983	2.224	4.003	<.001
	NCSV	8.425	6.380	11.125	<.001	3.918	2.655	5.782	<.001
	CSV	4.908	3.701	6.508	<.001	1.738	1.159	2.607	<.001
	Neglect	13.826	8.644	22.114	<.001	4.386	2.427	7.926	<.001

**Physical Violence**		Simple logistic regression				Multiple logistic regression			
$R^2_{Nagelkerke}$=.421		OR	99% CI		p	OR	99% CI		p
(multiple log reg)			Lower	Upper			Lower	Upper	
Gender	Male^O								
	Female	1.194	1.010	1.412	<.001	.663	.508	.865	<.001
Age	16 to 18 years^O								
	19 to 26 years	1.188	.855	1.650	.177	1.258	.824	1.922	.162
	27 to 40 years	.832	.599	1.155	.148	.946	.618	1.449	.738
	41 to 60 years	.412	.301	.564	<.001	.637	.421	.965	.005
	61 years	.160	.108	.236	<.001	.356	.212	.596	<.001
Athletic level	Recreational^O								
	Local	2.330	1.732	3.134	<.001	1.663	1.135	2.437	<.001
	Regional	3.551	2.626	4.802	<.001	2.075	1.398	3.082	<.001
	National	3.837	2.741	5.373	<.001	2.214	1.408	3.481	<.001
	International	5.645	3.696	8.622	<.001	3.152	1.787	5.559	<.001
Training time/week	< 2 hours^O								
	2.5 - 6 hours	1.970	1.566	2.479	<.001	1.344	.995	1.814	.011
	6.5 - 10 hours	3.470	2.621	4.594	<.001	1.542	1.059	2.245	.003
	10.5 - 14 hours	3.184	2.066	4.906	<.001	.995	.567	1.746	.982
	14.5 - 20 hours	4.470	2.433	8.214	<.001	1.416	.635	3.156	.264
	> 20 hours	5.483	2.423	12.405	<.001	1.934	.656	5.703	.116

**Physical Violence**		Simple logistic regression				Multiple logistic regression			
Early specialization	No[O]								
	Yes	2.131	1.706	2.661	<.001	1.402	1.047	1.876	.003
IV outside sports	Psych. violence	5.183	4.219	6.366	<.001	1.409	1.059	1.875	.002
	Physical violence	4.858	4.061	5.813	<.001	3.215	2.523	4.097	<.001
	NCSV	2.499	2.096	2.979	<.001	.913	.663	1.256	.461
	CSV	2.042	1.708	2.441	<.001	.934	.687	1.269	.564
	Neglect	3.711	2.744	5.020	<.001	1.124	.745	1.696	.464
IV inside sports	Psych. violence	7.528	6.011	9.427	<.001	3.038	2.274	4.059	<.001
	NCSV	4.331	3.568	5.258	<.001	1.830	1.379	2.429	<.001
	CSV	3.291	2.661	4.070	<.001	1.326	.977	1.799	.017
	Neglect	6.984	5.394	9.043	<.001	2.645	1.906	3.670	<.001

**NCSV**		Simple logistic regression				Multiple logistic regression			
$R^2_{Nagelkerke}$=.496		OR	99% CI		p	OR	99% CI		p
(multiple log reg)			Lower	Upper			Lower	Upper	
Gender	Male[O]								
	Female	3.746	3.076	4.561	<.001	1.633	1.209	2.207	<.001
Age	16 to 18 years[O]								
	19 to 26 years	1.443	1.018	2.045	.007	1.512	.920	2.485	.032
	27 to 40 years	1.139	.801	1.621	.340	1.518	.916	2.515	.033
	41 to 60 years	.648	.461	.910	.001	1.529	.927	2.521	.029
	61 years	.267	.173	.411	<.001	1.167	.614	2.218	.537
Athletic level	Recreational[O]								
	Local	1.325	1.732	3.134	.016	1.149	.754	1.752	.395
	Regional	1.738	1.281	2.359	<.001	1.173	.757	1.818	.349
	National	2.107	1.499	2.961	<.001	1.484	.895	2.460	.044
	International	2.231	1.447	3.437	<.001	1.382	.719	2.657	.202
Training time/week	< 2 hours[O]								
	2.5 - 6 hours	1.465	1.143	1.878	<.001	1.032	.729	1.461	.813
	6.5 - 10 hours	2.102	1.558	2.836	<.001	.992	.639	1.539	.962
	10.5 - 14 hours	2.815	1.814	4.369	<.001	1.429	.756	2.704	.149
	14.5 - 20 hours	2.473	1.317	4.643	<.001	1.047	.431	2.545	.893
	> 20 hours	3.504	1.561	7.864	<.001	1.628	.505	5.246	.283

**NCSV**		Simple logistic regression				Multiple logistic regression			
Early specialization	No[O]								
	Yes	1.379	1.088	1.749	<.001	.700	.499	.983	.007
IV outside sports	Psych. violence	5.639	4.411	7.210	<.001	1.261	.880	1.807	.097
	Physical violence	2.741	2.272	3.307	<.001	1.052	.785	1.411	.656
	NCSV	9.898	8.039	12.186	<.001	8.007	5.749	11.152	<.001
	CSV	3.803	3.139	4.608	<.001	.491	.351	.689	<.001
	Neglect	3.764	2.812	5.038	<.001	.848	.552	1.303	.323
IV inside sports	Psych. violence	8.425	6.380	11.125	<.001	3.986	2.708	5.869	<.001
	Physical violence	4.331	3.568	5.258	<.001	1.816	1.360	2.425	<.001
	CSV	8.755	6.978	10.985	<.001	4.156	3.057	5.651	<.001
	Neglect	5.925	4.662	7.530	<.001	1.802	1.281	2.537	<.001

**CSV**		Simple logistic regression				Multiple logistic regression			
$R^2_{Nagelkerke}$=.402		OR	99% CI		p	OR	99% CI		p
(multiple log reg)			Lower	Upper			Lower	Upper	
Gender	Male[O]								
	Female	4.794	3.789	6.067	<.001	2.519	1.827	3.473	<.001
Age	16 to 18 years[O]								
	19 to 26 years	1.155	.799	1.672	.313	1.046	.636	1.720	.817
	27 to 40 years	.788	.539	1.154	.107	.786	.470	1.313	.226
	41 to 60 years	.488	.338	.705	<.001	.827	.498	1.373	.334
	61 years	.270	.170	.429	<.001	.941	.495	1.787	.807
Athletic level	Recreational[O]								
	Local	1.417	1.009	1.992	.008	1.495	.955	2.342	.021
	Regional	1.768	1.251	2.498	<.001	1.565	.981	2.496	.013
	National	1.649	1.112	2.445	.001	1.072	.622	1.848	.741
	International	2.002	1.228	3.263	<.001	1.277	.642	2.541	.360
Training time/week	< 2 hours[O]								
	2.5 - 6 hours	1.465	1.143	1.878	<.001	1.292	.891	1.875	.076
	6.5 - 10 hours	2.102	1.558	2.836	<.001	1.386	.875	2.196	.068
	10.5 - 14 hours	2.815	1.814	4.369	<.001	1.058	.532	2.103	.832
	14.5 - 20 hours	2.473	1.317	4.643	<.001	1.682	.696	4.065	.129
	> 20 hours	3.504	1.561	7.864	<.001	2.199	.705	6.858	.074

**CSV**		Simple logistic regression				Multiple logistic regression			
Early specialization	No[O]								
	Yes	1.579	1.220	2.045	<.001	1.062	.755	1.496	.649
IV outside sports	Psych. violence	4.344	3.311	5.698	<.001	1.151	.785	1.687	.344
	Physical violence	2.305	1.871	2.838	<.001	1.023	.754	1.389	.846
	NCSV	5.030	4.047	6.251	<.001	.606	.422	.869	<.001
	CSV	7.390	5.908	9.244	<.001	4.744	3.403	6.613	<.001
	Neglect	4.219	3.136	5.676	<.001	1.178	.778	1.784	.309
IV inside sports	Psych. violence	4.908	3.701	6.508	<.001	1.730	1.158	2.584	<.001
	Physical violence	3.291	2,661	4,07	<.001	1.300	.955	1.769	.028
	NCSV	8.755	6.978	10.985	<.001	4.099	3.024	5.555	<.001
	Neglect	5.468	4.275	6.994	<.001	1.773	1.261	2.491	<.001

**Neglect**		Simple logistic regression				Multiple logistic regression			
$R^2_{Nagelkerke=}$.405		OR	99% CI		p	OR	99% CI		p
(multiple log reg)			Lower	Upper			Lower	Upper	
Gender	Male[O]								
	Female	2.352	1.859	2.977	<.001	1.100	.779	1.553	.478
Age	16 to 18 years[O]								
	19 to 26 years	.911	.62	1.338	.533	.762	.461	1.259	.163
	27 to 40 years	.647	.434	.963	.005	.676	.403	1.136	.052
	41 to 60 years	.360	.244	.532	<.001	.617	.366	1.039	.017
	61 years	.180	.106	.306	<.001	.618	.301	1.266	.084
Athletic level	Recreational[O]								
	Local	1.655	1.097	2.496	<.001	1.105	.652	1.871	.626
	Regional	2.034	1.342	3.084	<.001	1.085	.632	1.863	.697
	National	2.712	1.735	4.239	<.001	1.277	.703	2.320	.291
	International	3.487	2.052	5.928	<.001	1.127	.547	2.319	.671
Training time/week	< 2 hours[O]								
	2.5 - 6 hours	1.480	1.060	2.066	.003	.977	.640	1.489	.885
	6.5 - 10 hours	2.723	1.869	3.970	<.001	1.308	.798	2.144	.161
	10.5 - 14 hours	3.543	2.109	5.952	<.001	1.453	.734	2.876	.159
	14.5 - 20 hours	5.320	2.718	10.414	<.001	2.275	.958	5.404	.014
	> 20 hours	5.537	2.348	13.056	<.001	2.348	.775	7.116	.047

**Neglect**		Simple logistic regression				Multiple logistic regression			
Early specialization	No°								
	Yes	1.932	1.470	2.539	<.001	1.147	.805	1.633	.319
IV outside sports	Psych. violence	7.155	4.985	10.270	<.001	1.669	1.052	2.647	.004
	Physical violence	3.248	2.569	4.105	<.001	.914	.660	1.264	.473
	NCSV	4.202	3.319	5.319	<.001	1.029	.692	1.530	.855
	CSV	3.923	3.139	4.608	<.001	1.283	.880	1.871	.089
	Neglect	12.441	9.133	16.946	<.001	6.306	4.240	9.380	<.001
IV inside sports	Psych. violence	13.826	8.644	22.114	<.001	4.353	2.428	7.806	<.001
	Physical violence	6.984	5.394	9.043	<.001	2.542	1.832	3.528	<.001
	NCSV	3.764	2.812	5.038	<.001	1.811	1.289	2.546	<.001
	CSV	5.468	4.275	6.994	<.001	1.755	1.248	2.468	<.001

Note. OR = odd's ratio, CI = confidence interval, Psych. Violence = psychological violence, NCSV = non-contact sexual violence, CSV = contact sexual violence, ° = denotes reference category
